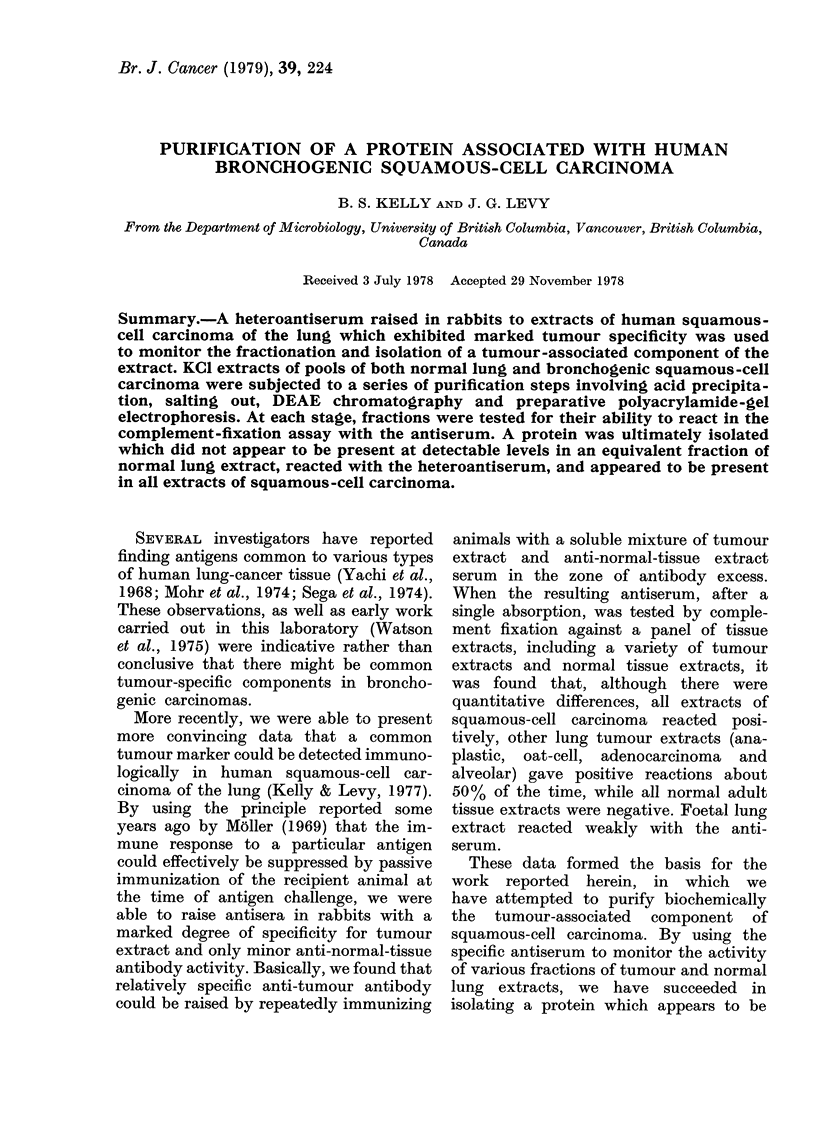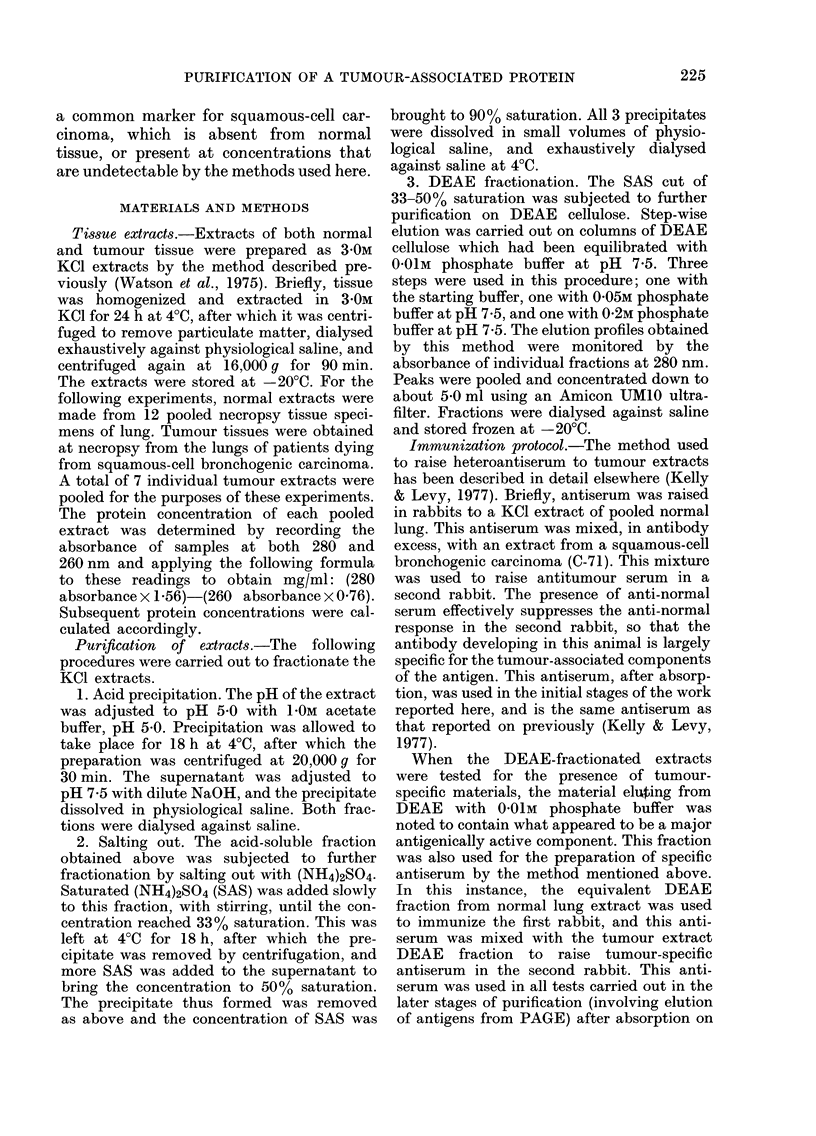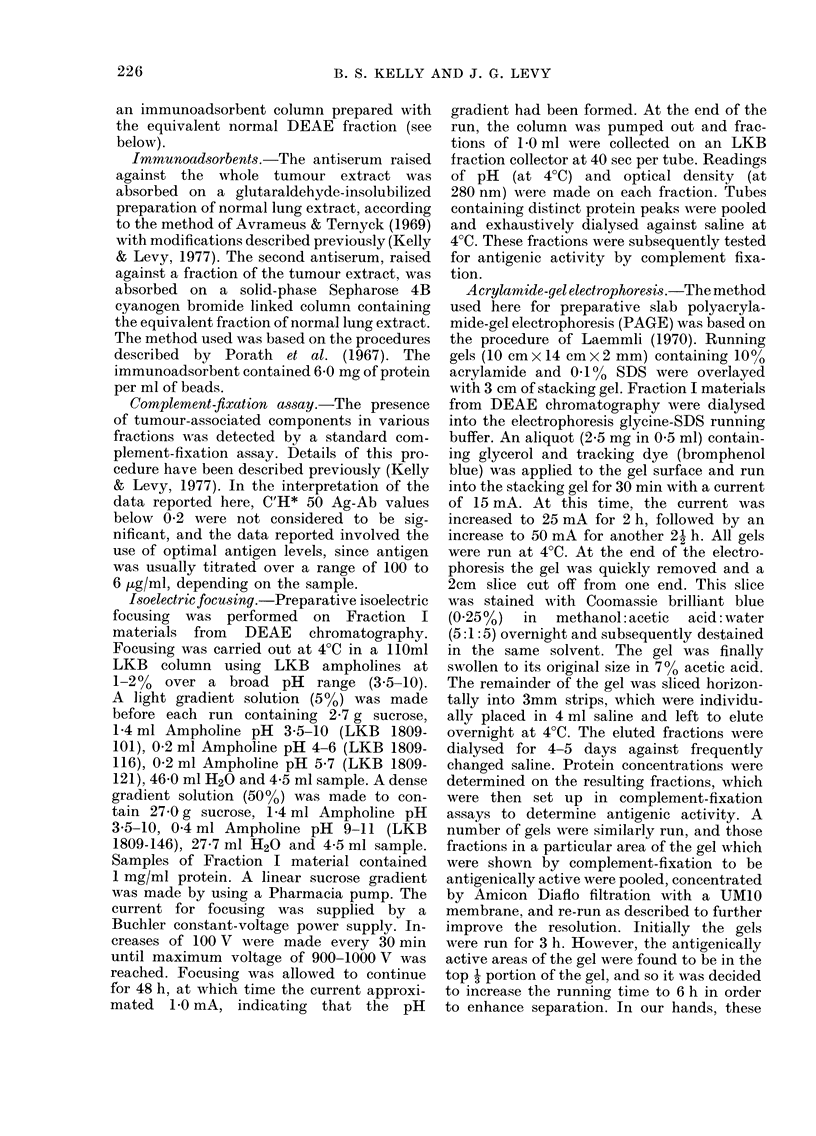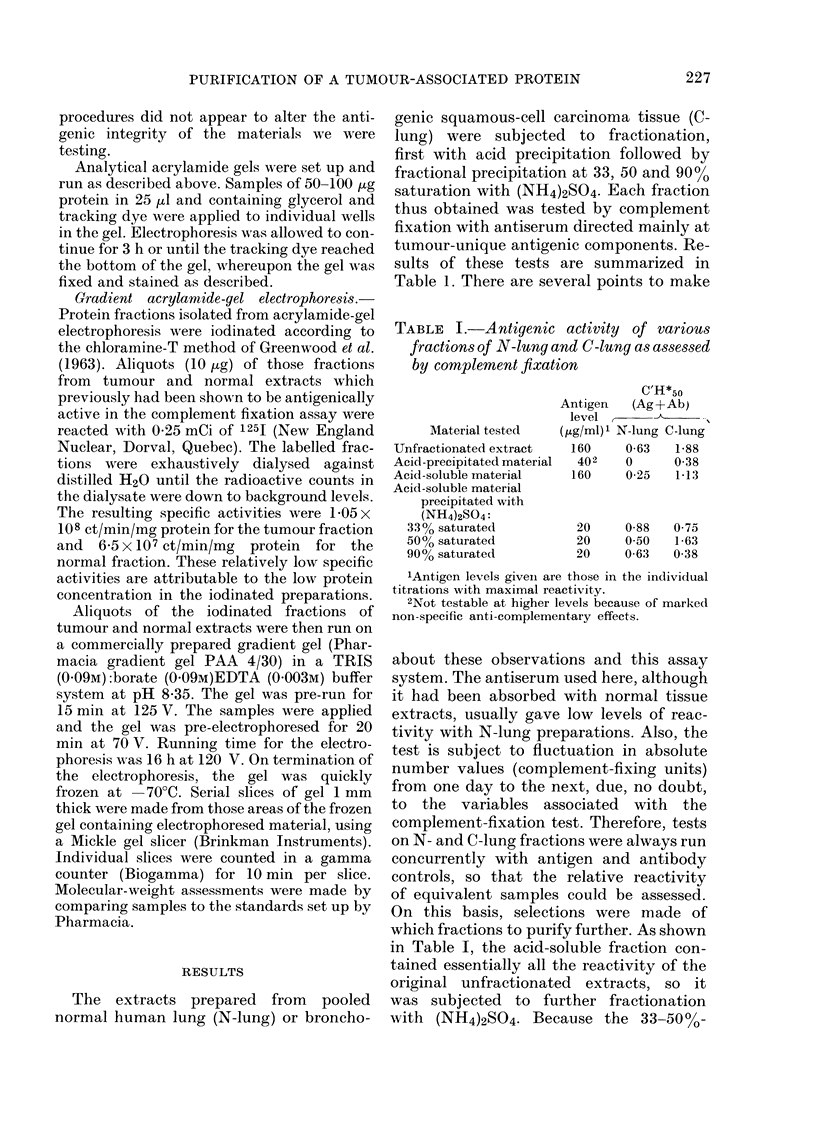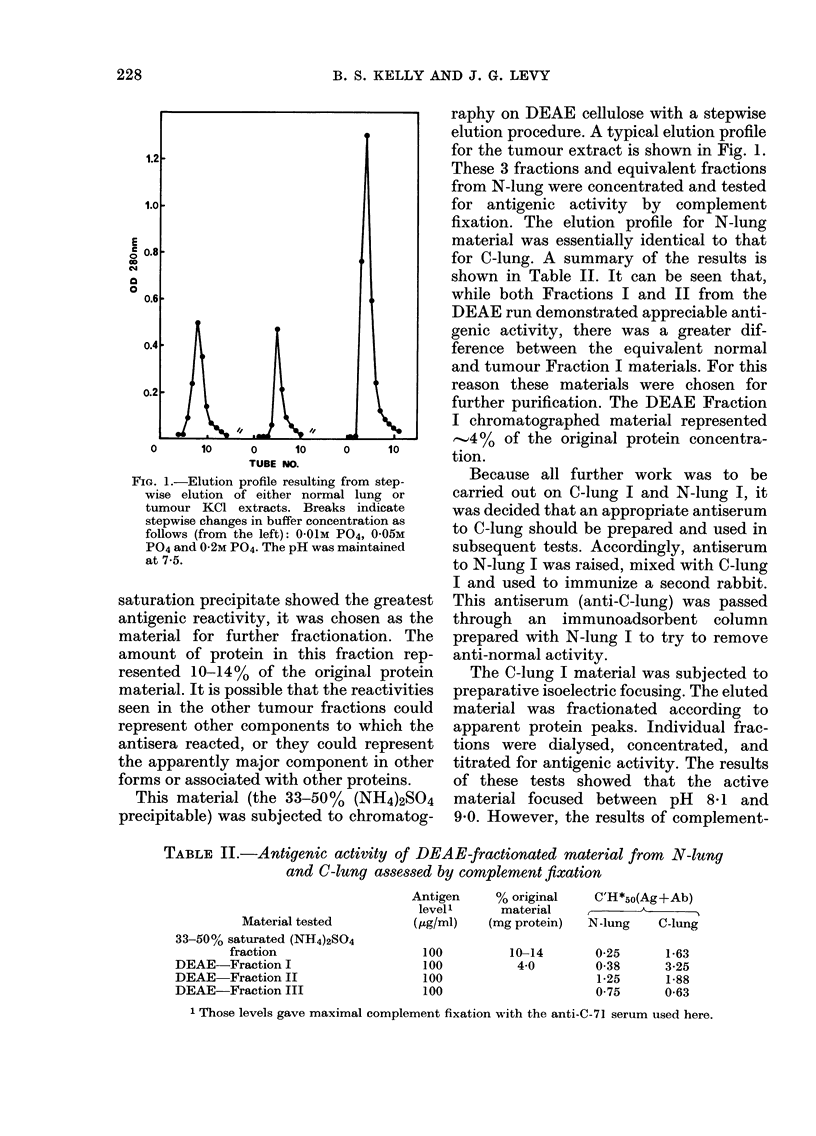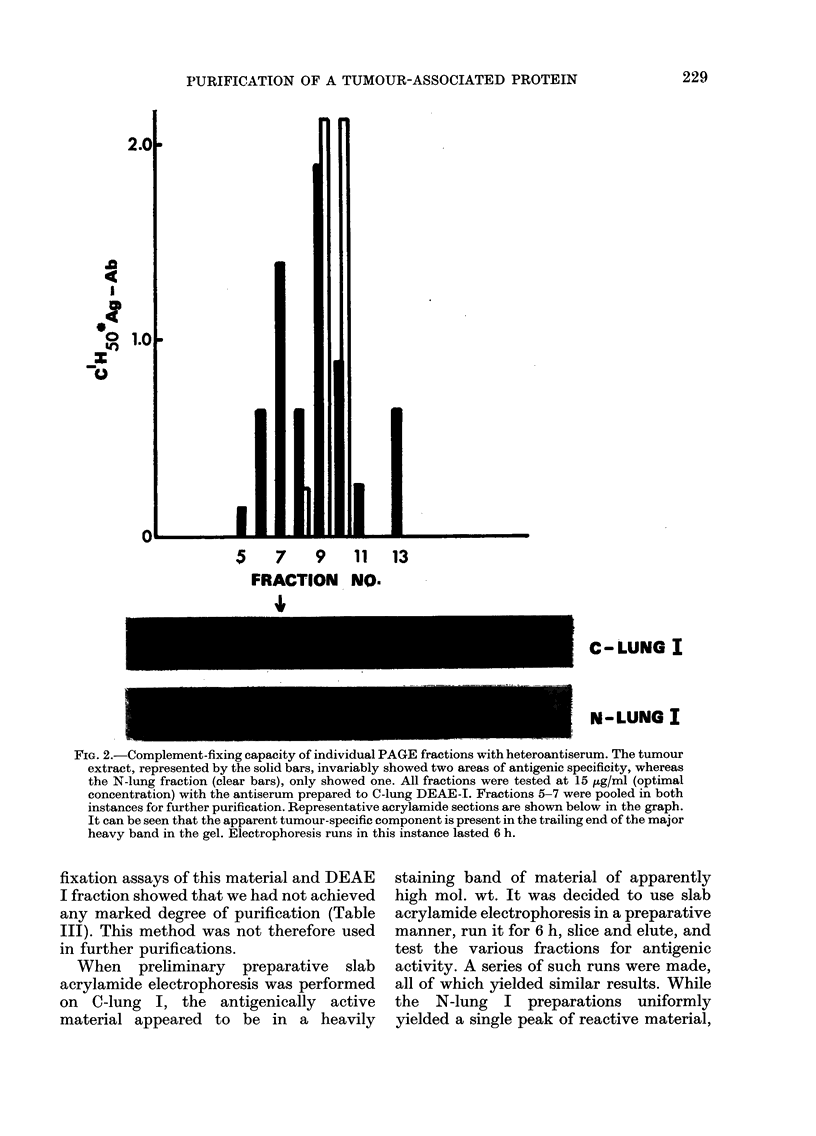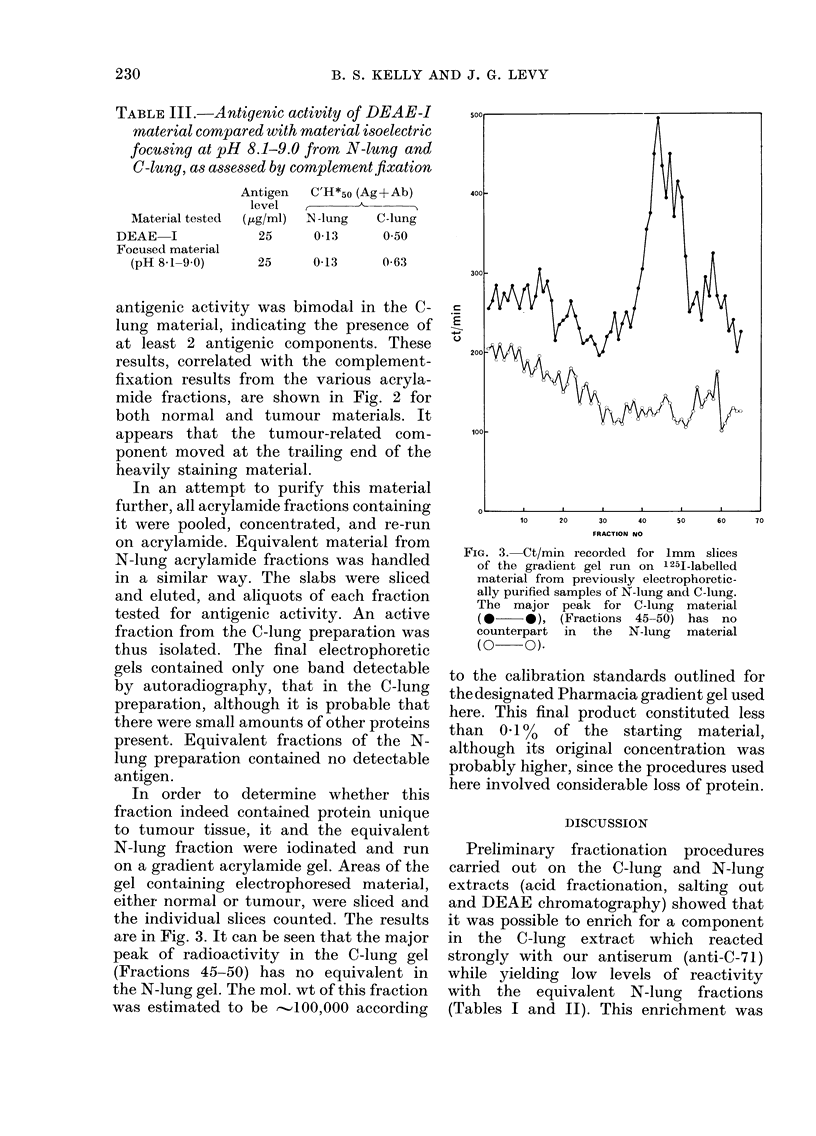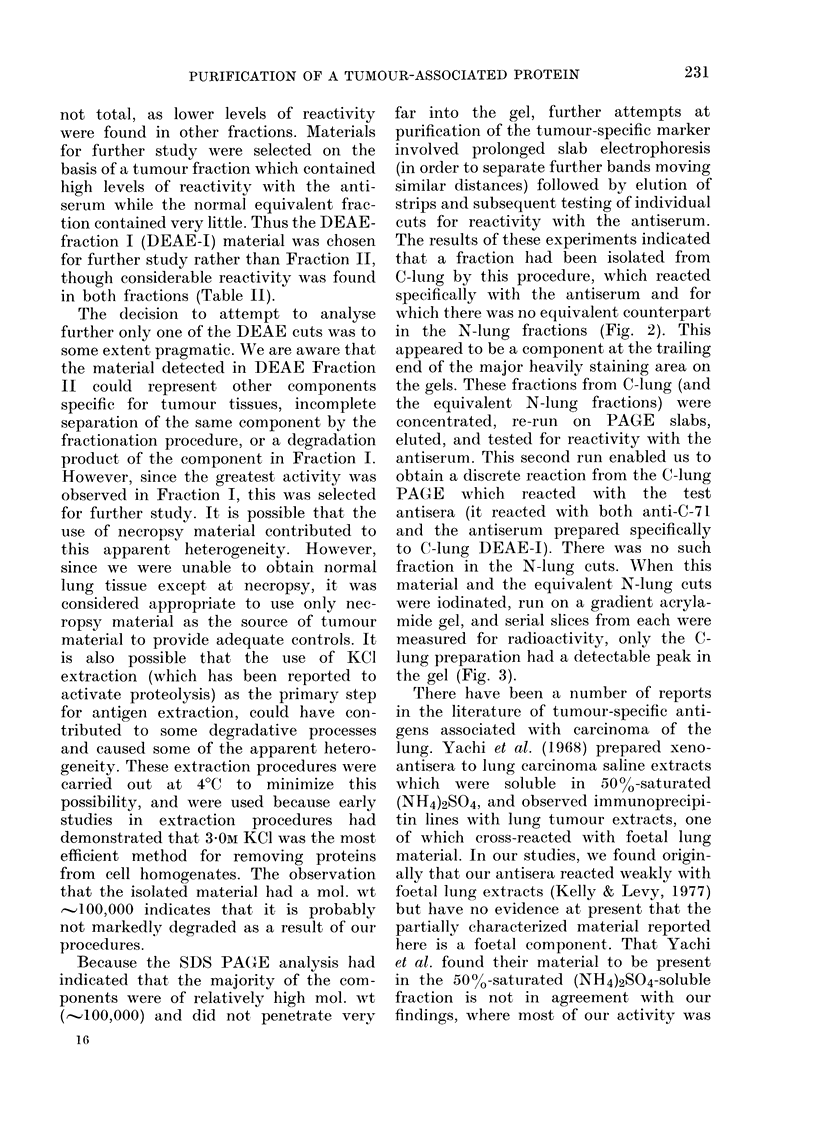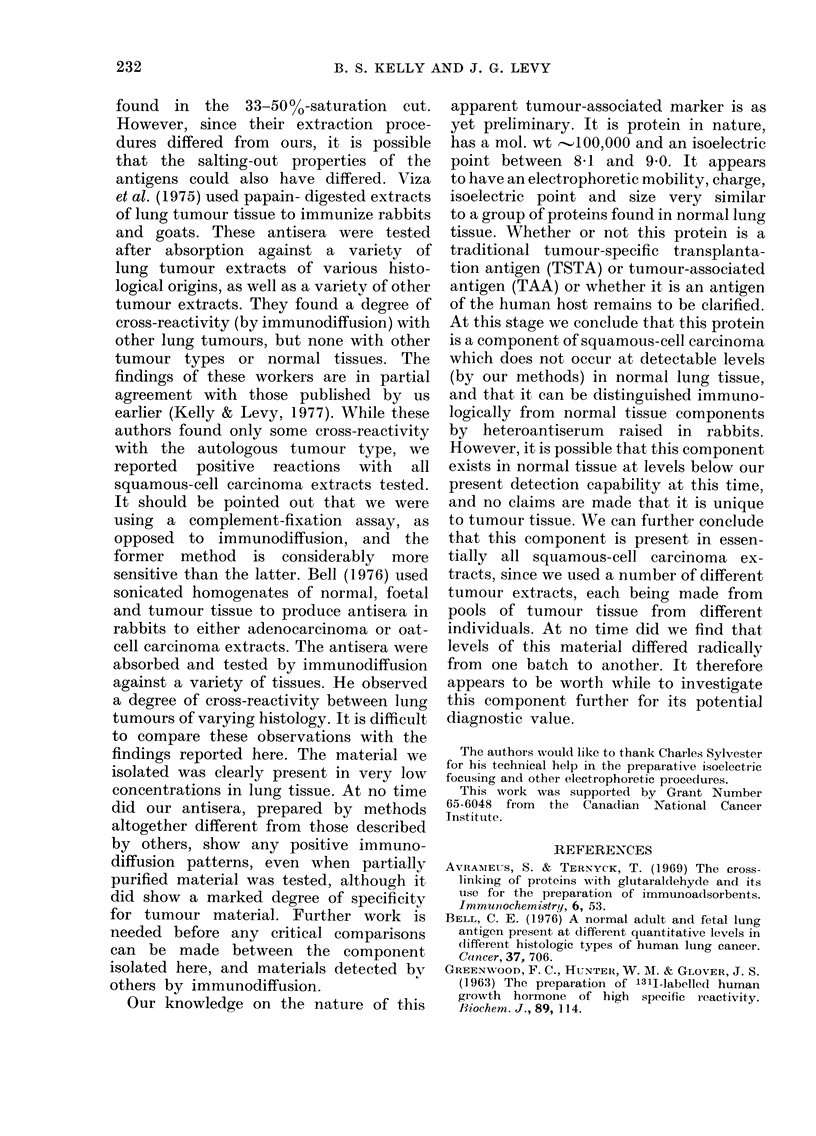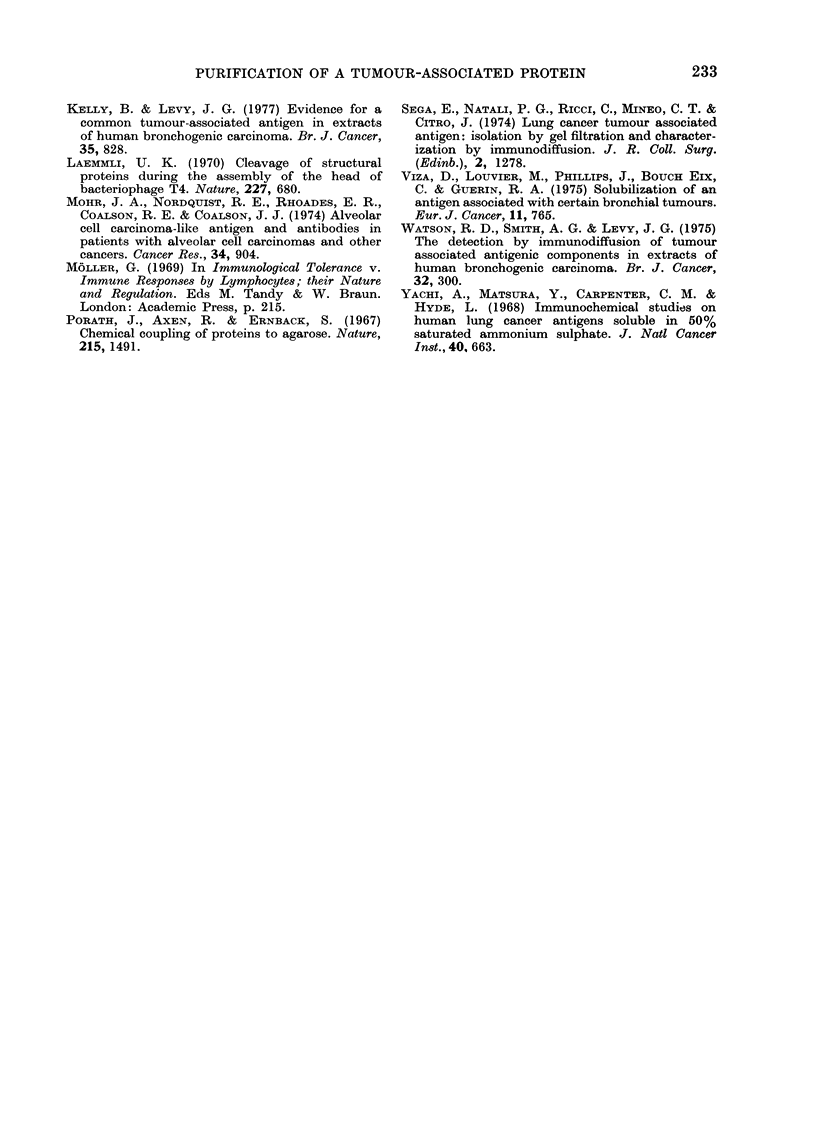# Purification of a protein associated with human bronchogenic squamous-cell carcinoma.

**DOI:** 10.1038/bjc.1979.45

**Published:** 1979-03

**Authors:** B. S. Kelly, J. G. Levy

## Abstract

**Images:**


*Br. J. Cancer* (1979), **39**, 224

### PURIFICATION OF A PROTEIN ASSOCIATED WITH HUMAN BRONCHOGENIC SQUAMOUS-CELL CARCINOMA

B. S. KELLY AND J. G. LEVY

*From the Department of Microbiology, University of British Columbia, Vancouver, British Columbia, Canada*

Received 3 July 1978 Accepted 29 November 1978

**Summary.—A heteroantiserum raised in rabbits to extracts of human squamous-cell carcinoma of the lung which exhibited marked tumour specificity was used to monitor the fractionation and isolation of a tumour-associated component of the extract. KCl extracts of pools of both normal lung and bronchogenic squamous-cell carcinoma were subjected to a series of purification steps involving acid precipitation, salting out, DEAE chromatography and preparative polyacrylamide-gel electrophoresis. At each stage, fractions were tested for their ability to react in the complement-fixation assay with the antiserum. A protein was ultimately isolated which did not appear to be present at detectable levels in an equivalent fraction of normal lung extract, reacted with the heteroantiserum, and appeared to be present in all extracts of squamous-cell carcinoma.**

SEVERAL investigators have reported finding antigens common to various types of human lung-cancer tissue (Yachi *et al.*, 1968; Mohr *et al.*, 1974; Sega *et al.*, 1974). These observations, as well as early work carried out in this laboratory (Watson *et al.*, 1975) were indicative rather than conclusive that there might be common tumour-specific components in bronchogenic carcinomas.

More recently, we were able to present more convincing data that a common tumour marker could be detected immunologically in human squamous-cell carcinoma of the lung (Kelly & Levy, 1977). By using the principle reported some years ago by Möller (1969) that the immune response to a particular antigen could effectively be suppressed by passive immunization of the recipient animal at the time of antigen challenge, we were able to raise antisera in rabbits with a marked degree of specificity for tumour extract and only minor anti-normal-tissue antibody activity. Basically, we found that relatively specific anti-tumour antibody could be raised by repeatedly immunizing animals with a soluble mixture of tumour extract and anti-normal-tissue extract serum in the zone of antibody excess. When the resulting antiserum, after a single absorption, was tested by complement fixation against a panel of tissue extracts, including a variety of tumour extracts and normal tissue extracts, it was found that, although there were quantitative differences, all extracts of squamous-cell carcinoma reacted positively, other lung tumour extracts (anaplastic, oat-cell, adenocarcinoma and alveolar) gave positive reactions about 50% of the time, while all normal adult tissue extracts were negative. Foetal lung extract reacted weakly with the antiserum.

These data formed the basis for the work reported herein, in which we have attempted to purify biochemically the tumour-associated component of squamous-cell carcinoma. By using the specific antiserum to monitor the activity of various fractions of tumour and normal lung extracts, we have succeeded in isolating a protein which appears to be a common marker for squamous-cell carcinoma, which is absent from normal tissue, or present at concentrations that are undetectable by the methods used here.

### MATERIALS AND METHODS

*Tissue extracts.*—Extracts of both normal and tumour tissue were prepared as 3·0M KCl extracts by the method described previously (Watson *et al.*, 1975). Briefly, tissue was homogenized and extracted in 3·0M KCl for 24 h at 4°C, after which it was centrifuged to remove particulate matter, dialysed exhaustively against physiological saline, and centrifuged again at 16,000 *g* for 90 min. The extracts were stored at −20°C. For the following experiments, normal extracts were made from 12 pooled necropsy tissue specimens of lung. Tumour tissues were obtained at necropsy from the lungs of patients dying from squamous-cell bronchogenic carcinoma. A total of 7 individual tumour extracts were pooled for the purposes of these experiments. The protein concentration of each pooled extract was determined by recording the absorbance of samples at both 280 and 260 nm and applying the following formula to these readings to obtain mg/ml: (280 absorbance × 1·56)—(260 absorbance × 0·76). Subsequent protein concentrations were calculated accordingly.

*Purification of extracts.*—The following procedures were carried out to fractionate the KCl extracts.

1. Acid precipitation. The pH of the extract was adjusted to pH 5·0 with 1·0M acetate buffer, pH 5·0. Precipitation was allowed to take place for 18 h at 4°C, after which the preparation was centrifuged at 20,000 *g* for 30 min. The supernatant was adjusted to pH 7·5 with dilute NaOH, and the precipitate dissolved in physiological saline. Both fractions were dialysed against saline.

2. Salting out. The acid-soluble fraction obtained above was subjected to further fractionation by salting out with $(NH_4)_2SO_4$. Saturated $(NH_4)_2SO_4$ (SAS) was added slowly to this fraction, with stirring, until the concentration reached 33% saturation. This was left at 4°C for 18 h, after which the precipitate was removed by centrifugation, and more SAS was added to the supernatant to bring the concentration to 50% saturation. The precipitate thus formed was removed as above and the concentration of SAS was brought to 90% saturation. All 3 precipitates were dissolved in small volumes of physiological saline, and exhaustively dialysed against saline at 4°C.

3. DEAE fractionation. The SAS cut of 33–50% saturation was subjected to further purification on DEAE cellulose. Step-wise elution was carried out on columns of DEAE cellulose which had been equilibrated with 0·01M phosphate buffer at pH 7·5. Three steps were used in this procedure; one with the starting buffer, one with 0·05M phosphate buffer at pH 7·5, and one with 0·2M phosphate buffer at pH 7·5. The elution profiles obtained by this method were monitored by the absorbance of individual fractions at 280 nm. Peaks were pooled and concentrated down to about 5·0 ml using an Amicon UM10 ultrafilter. Fractions were dialysed against saline and stored frozen at −20°C.

*Immunization protocol.*—The method used to raise heteroantiserum to tumour extracts has been described in detail elsewhere (Kelly & Levy, 1977). Briefly, antiserum was raised in rabbits to a KCl extract of pooled normal lung. This antiserum was mixed, in antibody excess, with an extract from a squamous-cell bronchogenic carcinoma (C-71). This mixture was used to raise antitumour serum in a second rabbit. The presence of anti-normal serum effectively suppresses the anti-normal response in the second rabbit, so that the antibody developing in this animal is largely specific for the tumour-associated components of the antigen. This antiserum, after absorption, was used in the initial stages of the work reported here, and is the same antiserum as that reported on previously (Kelly & Levy, 1977).

When the DEAE-fractionated extracts were tested for the presence of tumour-specific materials, the material eluting from DEAE with 0·01M phosphate buffer was noted to contain what appeared to be a major antigenically active component. This fraction was also used for the preparation of specific antiserum by the method mentioned above. In this instance, the equivalent DEAE fraction from normal lung extract was used to immunize the first rabbit, and this antiserum was mixed with the tumour extract DEAE fraction to raise tumour-specific antiserum in the second rabbit. This antiserum was used in all tests carried out in the later stages of purification (involving elution of antigens from PAGE) after absorption on an immunoadsorbent column prepared with the equivalent normal DEAE fraction (see below).

*Immunoadsorbents.*—The antiserum raised against the whole tumour extract was absorbed on a glutaraldehyde-insolubilized preparation of normal lung extract, according to the method of Avrameus & Ternyck (1969) with modifications described previously (Kelly & Levy, 1977). The second antiserum, raised against a fraction of the tumour extract, was absorbed on a solid-phase Sepharose 4B cyanogen bromide linked column containing the equivalent fraction of normal lung extract. The method used was based on the procedures described by Porath *et al.* (1967). The immunoadsorbent contained 6·0 mg of protein per ml of beads.

*Complement-fixation assay.*—The presence of tumour-associated components in various fractions was detected by a standard complement-fixation assay. Details of this procedure have been described previously (Kelly & Levy, 1977). In the interpretation of the data reported here, C'H* 50 Ag-Ab values below 0·2 were not considered to be significant, and the data reported involved the use of optimal antigen levels, since antigen was usually titrated over a range of 100 to 6 μg/ml, depending on the sample.

*Isoelectric focusing.*—Preparative isoelectric focusing was performed on Fraction I materials from DEAE chromatography. Focusing was carried out at 4°C in a 110ml LKB column using LKB ampholines at 1–2% over a broad pH range (3·5–10). A light gradient solution (5%) was made before each run containing 2·7 g sucrose, 1·4 ml Ampholine pH 3·5–10 (LKB 1809-101), 0·2 ml Ampholine pH 4–6 (LKB 1809-116), 0·2 ml Ampholine pH 5·7 (LKB 1809-121), 46·0 ml $H_2O$ and 4·5 ml sample. A dense gradient solution (50%) was made to contain 27·0 g sucrose, 1·4 ml Ampholine pH 3·5–10, 0·4 ml Ampholine pH 9–11 (LKB 1809-146), 27·7 ml $H_2O$ and 4·5 ml sample. Samples of Fraction I material contained 1 mg/ml protein. A linear sucrose gradient was made by using a Pharmacia pump. The current for focusing was supplied by a Buchler constant-voltage power supply. Increases of 100 V were made every 30 min until maximum voltage of 900–1000 V was reached. Focusing was allowed to continue for 48 h, at which time the current approximated 1·0 mA, indicating that the pH gradient had been formed. At the end of the run, the column was pumped out and fractions of 1·0 ml were collected on an LKB fraction collector at 40 sec per tube. Readings of pH (at 4°C) and optical density (at 280 nm) were made on each fraction. Tubes containing distinct protein peaks were pooled and exhaustively dialysed against saline at 4°C. These fractions were subsequently tested for antigenic activity by complement fixation.

*Acrylamide-gel electrophoresis.*—The method used here for preparative slab polyacrylamide-gel electrophoresis (PAGE) was based on the procedure of Laemmli (1970). Running gels (10 cm × 14 cm × 2 mm) containing 10% acrylamide and 0·1% SDS were overlayed with 3 cm of stacking gel. Fraction I materials from DEAE chromatography were dialysed into the electrophoresis glycine-SDS running buffer. An aliquot (2·5 mg in 0·5 ml) containing glycerol and tracking dye (bromphenol blue) was applied to the gel surface and run into the stacking gel for 30 min with a current of 15 mA. At this time, the current was increased to 25 mA for 2 h, followed by an increase to 50 mA for another $2\frac{1}{2}$ h. All gels were run at 4°C. At the end of the electrophoresis the gel was quickly removed and a 2cm slice cut off from one end. This slice was stained with Coomassie brilliant blue (0·25%) in methanol:acetic acid:water (5:1:5) overnight and subsequently destained in the same solvent. The gel was finally swollen to its original size in 7% acetic acid. The remainder of the gel was sliced horizontally into 3mm strips, which were individually placed in 4 ml saline and left to elute overnight at 4°C. The eluted fractions were dialysed for 4–5 days against frequently changed saline. Protein concentrations were determined on the resulting fractions, which were then set up in complement-fixation assays to determine antigenic activity. A number of gels were similarly run, and those fractions in a particular area of the gel which were shown by complement-fixation to be antigenically active were pooled, concentrated by Amicon Diaflo filtration with a UM10 membrane, and re-run as described to further improve the resolution. Initially the gels were run for 3 h. However, the antigenically active areas of the gel were found to be in the top $\frac{1}{3}$ portion of the gel, and so it was decided to increase the running time to 6 h in order to enhance separation. In our hands, these procedures did not appear to alter the antigenic integrity of the materials we were testing.

Analytical acrylamide gels were set up and run as described above. Samples of 50–100 $\mu$g protein in 25 $\mu$l and containing glycerol and tracking dye were applied to individual wells in the gel. Electrophoresis was allowed to continue for 3 h or until the tracking dye reached the bottom of the gel, whereupon the gel was fixed and stained as described.

*Gradient acrylamide-gel electrophoresis.*— Protein fractions isolated from acrylamide-gel electrophoresis were iodinated according to the chloramine-T method of Greenwood *et al.* (1963). Aliquots (10 $\mu$g) of those fractions from tumour and normal extracts which previously had been shown to be antigenically active in the complement fixation assay were reacted with 0·25 mCi of $^{125}$I (New England Nuclear, Dorval, Quebec). The labelled fractions were exhaustively dialysed against distilled $H_2O$ until the radioactive counts in the dialysate were down to background levels. The resulting specific activities were $1{\cdot}05 \times 10^8$ ct/min/mg protein for the tumour fraction and $6{\cdot}5 \times 10^7$ ct/min/mg protein for the normal fraction. These relatively low specific activities are attributable to the low protein concentration in the iodinated preparations.

Aliquots of the iodinated fractions of tumour and normal extracts were then run on a commercially prepared gradient gel (Pharmacia gradient gel PAA 4/30) in a TRIS (0·09M):borate (0·09M)EDTA (0·003M) buffer system at pH 8·35. The gel was pre-run for 15 min at 125 V. The samples were applied and the gel was pre-electrophoresed for 20 min at 70 V. Running time for the electrophoresis was 16 h at 120 V. On termination of the electrophoresis, the gel was quickly frozen at −70°C. Serial slices of gel 1 mm thick were made from those areas of the frozen gel containing electrophoresed material, using a Mickle gel slicer (Brinkman Instruments). Individual slices were counted in a gamma counter (Biogamma) for 10 min per slice. Molecular-weight assessments were made by comparing samples to the standards set up by Pharmacia.

### RESULTS

The extracts prepared from pooled normal human lung (N-lung) or bronchogenic squamous-cell carcinoma tissue (C-lung) were subjected to fractionation, first with acid precipitation followed by fractional precipitation at 33, 50 and 90% saturation with $(NH_4)_2SO_4$. Each fraction thus obtained was tested by complement fixation with antiserum directed mainly at tumour-unique antigenic components. Results of these tests are summarized in Table 1. There are several points to make

TABLE I.—*Antigenic activity of various fractions of N-lung and C-lung as assessed by complement fixation*

| Material tested | Antigen level ($\mu$g/ml)[1] | C′H*50 (Ag+Ab) N-lung | C-lung |
|---|---|---|---|
| Unfractionated extract | 160 | 0·63 | 1·88 |
| Acid-precipitated material | 40[2] | 0 | 0·38 |
| Acid-soluble material | 160 | 0·25 | 1·13 |
| Acid-soluble material precipitated with $(NH_4)_2SO_4$: | | | |
| 33% saturated | 20 | 0·88 | 0·75 |
| 50% saturated | 20 | 0·50 | 1·63 |
| 90% saturated | 20 | 0·63 | 0·38 |

[1]Antigen levels given are those in the individual titrations with maximal reactivity.

[2]Not testable at higher levels because of marked non-specific anti-complementary effects.

about these observations and this assay system. The antiserum used here, although it had been absorbed with normal tissue extracts, usually gave low levels of reactivity with N-lung preparations. Also, the test is subject to fluctuation in absolute number values (complement-fixing units) from one day to the next, due, no doubt, to the variables associated with the complement-fixation test. Therefore, tests on N- and C-lung fractions were always run concurrently with antigen and antibody controls, so that the relative reactivity of equivalent samples could be assessed. On this basis, selections were made of which fractions to purify further. As shown in Table I, the acid-soluble fraction contained essentially all the reactivity of the original unfractionated extracts, so it was subjected to further fractionation with $(NH_4)_2SO_4$. Because the 33–50%-

FIG. 1.—Elution profile resulting from stepwise elution of either normal lung or tumour KCl extracts. Breaks indicate stepwise changes in buffer concentration as follows (from the left): 0·01M $PO_4$, 0·05M $PO_4$ and 0·2M $PO_4$. The pH was maintained at 7·5.

saturation precipitate showed the greatest antigenic reactivity, it was chosen as the material for further fractionation. The amount of protein in this fraction represented 10–14% of the original protein material. It is possible that the reactivities seen in the other tumour fractions could represent other components to which the antisera reacted, or they could represent the apparently major component in other forms or associated with other proteins.

This material (the 33–50% $(NH_4)_2SO_4$ precipitable) was subjected to chromatography on DEAE cellulose with a stepwise elution procedure. A typical elution profile for the tumour extract is shown in Fig. 1. These 3 fractions and equivalent fractions from N-lung were concentrated and tested for antigenic activity by complement fixation. The elution profile for N-lung material was essentially identical to that for C-lung. A summary of the results is shown in Table II. It can be seen that, while both Fractions I and II from the DEAE run demonstrated appreciable antigenic activity, there was a greater difference between the equivalent normal and tumour Fraction I materials. For this reason these materials were chosen for further purification. The DEAE Fraction I chromatographed material represented ~4% of the original protein concentration.

Because all further work was to be carried out on C-lung I and N-lung I, it was decided that an appropriate antiserum to C-lung should be prepared and used in subsequent tests. Accordingly, antiserum to N-lung I was raised, mixed with C-lung I and used to immunize a second rabbit. This antiserum (anti-C-lung) was passed through an immunoadsorbent column prepared with N-lung I to try to remove anti-normal activity.

The C-lung I material was subjected to preparative isoelectric focusing. The eluted material was fractionated according to apparent protein peaks. Individual fractions were dialysed, concentrated, and titrated for antigenic activity. The results of these tests showed that the active material focused between pH 8·1 and 9·0. However, the results of complement-

TABLE II.—*Antigenic activity of DEAE-fractionated material from N-lung and C-lung assessed by complement fixation*

| Material tested | Antigen level[1] ($\mu$g/ml) | % original material (mg protein) | C′H*50(Ag+Ab) N-lung | C′H*50(Ag+Ab) C-lung |
|---|---|---|---|---|
| 33–50% saturated $(NH_4)_2SO_4$ fraction | 100 | 10–14 | 0·25 | 1·63 |
| DEAE—Fraction I | 100 | 4·0 | 0·38 | 3·25 |
| DEAE—Fraction II | 100 | | 1·25 | 1·88 |
| DEAE—Fraction III | 100 | | 0·75 | 0·63 |

[1] Those levels gave maximal complement fixation with the anti-C-71 serum used here.

FIG. 2.—Complement-fixing capacity of individual PAGE fractions with heteroantiserum. The tumour extract, represented by the solid bars, invariably showed two areas of antigenic specificity, whereas the N-lung fraction (clear bars), only showed one. All fractions were tested at 15 $\mu$g/ml (optimal concentration) with the antiserum prepared to C-lung DEAE-I. Fractions 5–7 were pooled in both instances for further purification. Representative acrylamide sections are shown below in the graph. It can be seen that the apparent tumour-specific component is present in the trailing end of the major heavy band in the gel. Electrophoresis runs in this instance lasted 6 h.

fixation assays of this material and DEAE I fraction showed that we had not achieved any marked degree of purification (Table III). This method was not therefore used in further purifications.

When preliminary preparative slab acrylamide electrophoresis was performed on C-lung I, the antigenically active material appeared to be in a heavily staining band of material of apparently high mol. wt. It was decided to use slab acrylamide electrophoresis in a preparative manner, run it for 6 h, slice and elute, and test the various fractions for antigenic activity. A series of such runs were made, all of which yielded similar results. While the N-lung I preparations uniformly yielded a single peak of reactive material,

TABLE III.—*Antigenic activity of DEAE-I material compared with material isoelectric focusing at pH 8.1–9.0 from N-lung and C-lung, as assessed by complement fixation*

| Material tested | Antigen level (μg/ml) | C'H*50 (Ag+Ab) N-lung | C-lung |
|---|---|---|---|
| DEAE—I | 25 | 0·13 | 0·50 |
| Focused material (pH 8·1–9·0) | 25 | 0·13 | 0·63 |

antigenic activity was bimodal in the C-lung material, indicating the presence of at least 2 antigenic components. These results, correlated with the complement-fixation results from the various acrylamide fractions, are shown in Fig. 2 for both normal and tumour materials. It appears that the tumour-related component moved at the trailing end of the heavily staining material.

In an attempt to purify this material further, all acrylamide fractions containing it were pooled, concentrated, and re-run on acrylamide. Equivalent material from N-lung acrylamide fractions was handled in a similar way. The slabs were sliced and eluted, and aliquots of each fraction tested for antigenic activity. An active fraction from the C-lung preparation was thus isolated. The final electrophoretic gels contained only one band detectable by autoradiography, that in the C-lung preparation, although it is probable that there were small amounts of other proteins present. Equivalent fractions of the N-lung preparation contained no detectable antigen.

In order to determine whether this fraction indeed contained protein unique to tumour tissue, it and the equivalent N-lung fraction were iodinated and run on a gradient acrylamide gel. Areas of the gel containing electrophoresed material, either normal or tumour, were sliced and the individual slices counted. The results are in Fig. 3. It can be seen that the major peak of radioactivity in the C-lung gel (Fractions 45–50) has no equivalent in the N-lung gel. The mol. wt of this fraction was estimated to be ~100,000 according

FIG. 3.—Ct/min recorded for 1mm slices of the gradient gel run on $^{125}$I-labelled material from previously electrophoretically purified samples of N-lung and C-lung. The major peak for C-lung material (●——●), (Fractions 45–50) has no counterpart in the N-lung material (○——○).

to the calibration standards outlined for the designated Pharmacia gradient gel used here. This final product constituted less than 0·1% of the starting material, although its original concentration was probably higher, since the procedures used here involved considerable loss of protein.

### DISCUSSION

Preliminary fractionation procedures carried out on the C-lung and N-lung extracts (acid fractionation, salting out and DEAE chromatography) showed that it was possible to enrich for a component in the C-lung extract which reacted strongly with our antiserum (anti-C-71) while yielding low levels of reactivity with the equivalent N-lung fractions (Tables I and II). This enrichment was not total, as lower levels of reactivity were found in other fractions. Materials for further study were selected on the basis of a tumour fraction which contained high levels of reactivity with the antiserum while the normal equivalent fraction contained very little. Thus the DEAE-fraction I (DEAE-I) material was chosen for further study rather than Fraction II, though considerable reactivity was found in both fractions (Table II).

The decision to attempt to analyse further only one of the DEAE cuts was to some extent pragmatic. We are aware that the material detected in DEAE Fraction II could represent other components specific for tumour tissues, incomplete separation of the same component by the fractionation procedure, or a degradation product of the component in Fraction I. However, since the greatest activity was observed in Fraction I, this was selected for further study. It is possible that the use of necropsy material contributed to this apparent heterogeneity. However, since we were unable to obtain normal lung tissue except at necropsy, it was considered appropriate to use only necropsy material as the source of tumour material to provide adequate controls. It is also possible that the use of KCl extraction (which has been reported to activate proteolysis) as the primary step for antigen extraction, could have contributed to some degradative processes and caused some of the apparent heterogeneity. These extraction procedures were carried out at 4°C to minimize this possibility, and were used because early studies in extraction procedures had demonstrated that 3·0M KCl was the most efficient method for removing proteins from cell homogenates. The observation that the isolated material had a mol. wt ~100,000 indicates that it is probably not markedly degraded as a result of our procedures.

Because the SDS PAGE analysis had indicated that the majority of the components were of relatively high mol. wt (~100,000) and did not penetrate very far into the gel, further attempts at purification of the tumour-specific marker involved prolonged slab electrophoresis (in order to separate further bands moving similar distances) followed by elution of strips and subsequent testing of individual cuts for reactivity with the antiserum. The results of these experiments indicated that a fraction had been isolated from C-lung by this procedure, which reacted specifically with the antiserum and for which there was no equivalent counterpart in the N-lung fractions (Fig. 2). This appeared to be a component at the trailing end of the major heavily staining area on the gels. These fractions from C-lung (and the equivalent N-lung fractions) were concentrated, re-run on PAGE slabs, eluted, and tested for reactivity with the antiserum. This second run enabled us to obtain a discrete reaction from the C-lung PAGE which reacted with the test antisera (it reacted with both anti-C-71 and the antiserum prepared specifically to C-lung DEAE-I). There was no such fraction in the N-lung cuts. When this material and the equivalent N-lung cuts were iodinated, run on a gradient acrylamide gel, and serial slices from each were measured for radioactivity, only the C-lung preparation had a detectable peak in the gel (Fig. 3).

There have been a number of reports in the literature of tumour-specific antigens associated with carcinoma of the lung. Yachi *et al.* (1968) prepared xenoantisera to lung carcinoma saline extracts which were soluble in 50%-saturated $(NH_4)_2SO_4$, and observed immunoprecipitin lines with lung tumour extracts, one of which cross-reacted with foetal lung material. In our studies, we found originally that our antisera reacted weakly with foetal lung extracts (Kelly & Levy, 1977) but have no evidence at present that the partially characterized material reported here is a foetal component. That Yachi *et al.* found their material to be present in the 50%-saturated $(NH_4)_2SO_4$-soluble fraction is not in agreement with our findings, where most of our activity was found in the 33–50%-saturation cut. However, since their extraction procedures differed from ours, it is possible that the salting-out properties of the antigens could also have differed. Viza *et al.* (1975) used papain- digested extracts of lung tumour tissue to immunize rabbits and goats. These antisera were tested after absorption against a variety of lung tumour extracts of various histological origins, as well as a variety of other tumour extracts. They found a degree of cross-reactivity (by immunodiffusion) with other lung tumours, but none with other tumour types or normal tissues. The findings of these workers are in partial agreement with those published by us earlier (Kelly & Levy, 1977). While these authors found only some cross-reactivity with the autologous tumour type, we reported positive reactions with all squamous-cell carcinoma extracts tested. It should be pointed out that we were using a complement-fixation assay, as opposed to immunodiffusion, and the former method is considerably more sensitive than the latter. Bell (1976) used sonicated homogenates of normal, foetal and tumour tissue to produce antisera in rabbits to either adenocarcinoma or oat-cell carcinoma extracts. The antisera were absorbed and tested by immunodiffusion against a variety of tissues. He observed a degree of cross-reactivity between lung tumours of varying histology. It is difficult to compare these observations with the findings reported here. The material we isolated was clearly present in very low concentrations in lung tissue. At no time did our antisera, prepared by methods altogether different from those described by others, show any positive immunodiffusion patterns, even when partially purified material was tested, although it did show a marked degree of specificity for tumour material. Further work is needed before any critical comparisons can be made between the component isolated here, and materials detected by others by immunodiffusion.

Our knowledge on the nature of this apparent tumour-associated marker is as yet preliminary. It is protein in nature, has a mol. wt $\sim$100,000 and an isoelectric point between 8·1 and 9·0. It appears to have an electrophoretic mobility, charge, isoelectric point and size very similar to a group of proteins found in normal lung tissue. Whether or not this protein is a traditional tumour-specific transplantation antigen (TSTA) or tumour-associated antigen (TAA) or whether it is an antigen of the human host remains to be clarified. At this stage we conclude that this protein is a component of squamous-cell carcinoma which does not occur at detectable levels (by our methods) in normal lung tissue, and that it can be distinguished immunologically from normal tissue components by heteroantiserum raised in rabbits. However, it is possible that this component exists in normal tissue at levels below our present detection capability at this time, and no claims are made that it is unique to tumour tissue. We can further conclude that this component is present in essentially all squamous-cell carcinoma extracts, since we used a number of different tumour extracts, each being made from pools of tumour tissue from different individuals. At no time did we find that levels of this material differed radically from one batch to another. It therefore appears to be worth while to investigate this component further for its potential diagnostic value.

The authors would like to thank Charles Sylvester for his technical help in the preparative isoelectric focusing and other electrophoretic procedures.

This work was supported by Grant Number 65-6048 from the Canadian National Cancer Institute.

### REFERENCES

AVRAMEUS, S. & TERNYCK, T. (1969) The cross-linking of proteins with glutaraldehyde and its use for the preparation of immunoadsorbents. *Immunochemistry*, **6**, 53.

BELL, C. E. (1976) A normal adult and fetal lung antigen present at different quantitative levels in different histologic types of human lung cancer. *Cancer*, **37**, 706.

GREENWOOD, F. C., HUNTER, W. M. & GLOVER, J. S. (1963) The preparation of $^{131}$I-labelled human growth hormone of high specific reactivity. *Biochem. J.*, **89**, 114.

KELLY, B. & LEVY, J. G. (1977) Evidence for a common tumour-associated antigen in extracts of human bronchogenic carcinoma. *Br. J. Cancer*, **35**, 828.

LAEMMLI, U. K. (1970) Cleavage of structural proteins during the assembly of the head of bacteriophage T4. *Nature*, **227**, 680.

MOHR, J. A., NORDQUIST, R. E., RHOADES, E. R., COALSON, R. E. & COALSON, J. J. (1974) Alveolar cell carcinoma-like antigen and antibodies in patients with alveolar cell carcinomas and other cancers. *Cancer Res.*, **34**, 904.

MÖLLER, G. (1969) In *Immunological Tolerance* v. *Immune Responses by Lymphocytes; their Nature and Regulation*. Eds M. Tandy & W. Braun. London: Academic Press, p. 215.

PORATH, J., AXEN, R. & ERNBACK, S. (1967) Chemical coupling of proteins to agarose. *Nature*, **215**, 1491.

SEGA, E., NATALI, P. G., RICCI, C., MINEO, C. T. & CITRO, J. (1974) Lung cancer tumour associated antigen: isolation by gel filtration and characterization by immunodiffusion. *J. R. Coll. Surg.* (*Edinb.*), **2**, 1278.

VIZA, D., LOUVIER, M., PHILLIPS, J., BOUCH EIX, C. & GUERIN, R. A. (1975) Solubilization of an antigen associated with certain bronchial tumours. *Eur. J. Cancer*, **11**, 765.

WATSON, R. D., SMITH, A. G. & LEVY, J. G. (1975) The detection by immunodiffusion of tumour associated antigenic components in extracts of human bronchogenic carcinoma. *Br. J. Cancer*, **32**, 300.

YACHI, A., MATSURA, Y., CARPENTER, C. M. & HYDE, L. (1968) Immunochemical studies on human lung cancer antigens soluble in 50% saturated ammonium sulphate. *J. Natl Cancer Inst.*, **40**, 663.